# Axillary lymph node dissection on the run?

**Published:** 2017-03

**Authors:** N Maeseele, J Faes, T Van de Putte, J Vlasselaer, E de Jonge, JC Schobbens, K Deraedt, G Debrock, G Van de Putte

**Affiliations:** Ziekenhuis Oost Limburg, Multidisciplinary Breast Clinic, Schiepse Bos 6, 3600 Genk

**Keywords:** Sentinel node, nomogram, completion axillary lymph node dissection, breast carcinoma, outcome, morbidity

## Abstract

The standard approach of performing a completion axillary lymph node dissection (cALND) after a positive sentinel node for breast cancer patients is no longer generally accepted. This study applied the criterion of a 27% risk of having residual positive lymph nodes calculated by the MD Anderson nomogram to perform a cALND. This 27% cut-off is based on the number of positive non-sentinels in the Z0011 trial. A cohort of 166 cN0, sentinel positive breast cancer patients was used to validate the MD Anderson nomogram. ROC (Receiver Operating Characteristic) analysis shows an AUC (Area Under the Curve) of 0.76 and an optimal cut-off at 34% risk of positive non- SLNs (sensitivity 86%, specificity 57%). The 27% cut-off has a sensitivity of 88% and a specificity of 41% to detect positive non-sentinels. In a second cohort (N= 114) the 27% cut-off criterion was prospectively applied and appeared to be practice changing. Although we take minimal risk to leave disease behind (2/166 patients >3 positive nodes), 30.7 % in the first cohort and 54.4 % of the patients in the second cohort could be spared a cALND. The Z0011 criteria would have had more impact, omitting 90% of the cALND, but leaves more disease behind. The impact of leaving disease behind on survival remains unanswered but is awaited by long term follow up of large prospective cohort studies.

## Introduction

In the past years, the standard surgical approach to the axilla in breast cancer has changed from axillary lymph node dissection (ALND) to sentinel node biopsy (SNB) because of similar overall survival (Krag et al., 2010), less arm morbidity and better quality of life (Mansel et al., 2006). Current guidelines recommend completion ALND (cALND) if the sentinel lymph node (SLN) contains metastasis. However, some patients with positive SLNs may be managed without completion ALND. In 2010 a randomized study by the American College of Surgeons Oncology Group (ACOSOG Z0011 trial) showed that a cALND in patients with 1-2 positive SLNs did not alter the local recurrence rate, nor survival (Giuliano et al., 2010). This trial led many to question the role of cALND as the gold standard in women with a positive SNB. Several nomograms to calculate the risk of positive non-SLNs became available and over time more and more breast units implemented a strategy to decrease their cALND rate in their patient population (Van den Hoven et al., 2015). However, one has to recognize the pitfalls in extrapolating the results from the ACOSOG Z0011 trial based on a population at low risk for axillary lymph node involvement to other cohorts with a different disease profile. Therefore the aim of this study was twofold: firstly to validate the MD Anderson nomogram in predicting affected non-SLNs in our population and to evaluate the performance of a cut-off of 27%, 27% being the proportion of women in the Z0011 trial with positive non-SLNs, in reducing the number of unnecessary cALNDs in our population. Secondly, to prospectively test the use of this cut-off in a second cohort.

## Materials and methods

Data of breast cancer patients with a N0 status both clinically and ultrasonographically, showing a positive SLN and submitted to a cALND between 2002 and 2011 were used to validate the MD Anderson nomogram. Patients who received neoadjuvant chemotherapy were excluded from this study. For the validation of the nomogram a ROC (receiver operating characteristic) analysis was performed. We also tested the performance of a 27% cut-off defined as having a risk of positive non-SLNs of 27% or less. This percentage was the number of patients with positive non-SLNs in the cALND arm of the randomized Z0011 trial. It represents an estimation of the population for which the study findings are proven. To calculate the risk of positive non-SLNs for each patient we used the MD Anderson nomogram. This nomogram is available through http://www3.MDAnderson.org/app/medcalc/bc_nomogram2 (Mittendorf et al., 2012). The parameters used in the model are tumour histology and size, the number of SLN’s removed and the number of positive SLN’s, the maximum size of nodal metastasis, presence of lymphovascular invasion and extra nodal extension. Since residual positive nodes can be considered a risk for recurrence, the performance of the 27% cut-off was measured by its ability to avoid residual positive lymph nodes while still reducing the number of cALND. The performance of the 27% cut-off value was prospectively tested in another cohort of N0 patients between 2012 and 2015 who showed a positive SLN. A cALND was performed only if the patient had a risk of positive non-SLNs exceeding the cut-off of 27% according the MD Anderson nomogram. All patients received radiotherapy to extended fields as described by Jagsi et al. (2014). Patients treated with mastectomy and SNB were also included, since a positive SLN indicates radiotherapy in our management protocol. Comparisons of categorical data were made by the chi-square method, and of continuous data by the student-t test.

## Results

**Figure 1:**
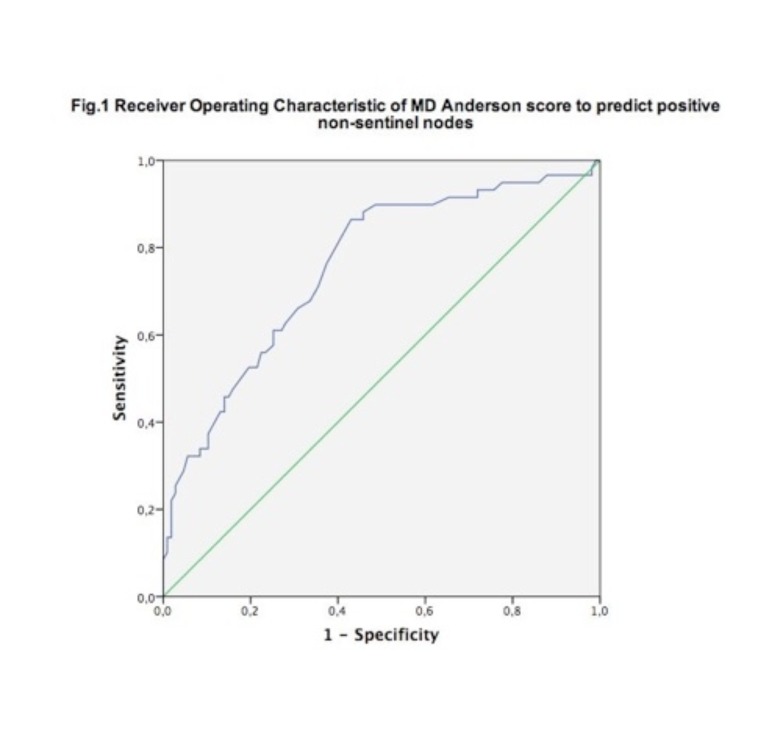
— Receiver Operating Characteristic of MD Anderson score to predict positive non-sentinel nodes.

Subject of the validation study was a cohort of 166 consecutive breast cancer patients. In the prospective study we registered a total of 114 breast cancer patients. The demographic and clinical characteristics of the patients in both populations are summarized in Table I. There is a significant difference between the two populations for age and tumour grade. Grade 3 tumours were observed more frequent in the first cohort (31.1% vs. 18.4%). The second cohort has a higher mean age (56.2 vs. 60.8). The clinical stage, tumour size, hormonal status or lymphovascular invasion were not statistically different. Regarding the lymph nodes, patients in the second cohort had more SLNs removed and the mean number of positive SLNs per patient is higher. The total number of positive nodes is not comparable because not all patients in the second cohort had a cALND. The percentage of nodal micro- and macro- metastases does not differ significantly between the two groups. The validation part of this study evaluated the prediction of the risk of positive SLNs by the MD Anderson nomogram. Validation of the MD Anderson nomogram by ROC analysis showed an area under the curve (AUC) of 0.76 and an optimal cut-off at 34% risk of positive non-SLNs. (Fig. 1) The 34% cut-off has a sensitivity of 86% and a specificity of 57%. The 27% cut-off has a sensitivity of 88% and a specificity of 41%. Of the 166 patients in the first cohort, 51 (30.7%) had a MDA score ≤ 27%. In these patients a cALND could have been avoided. A total of 59/166 had positive non-SLNs (35,5 %). Of these 59 patients, 52 had a MDA score of >27% and thus residual positive nonSLNs in these women would have been picked up by a cALND (sensitivity 88%). A total of 107/166 patients (64.5%) did not have positive non-SLNs and 44 of them had a MDA score ≤ 27%. This translates to 44 cases that correctly would have been spared a cALND (specificity 41%). Table II also represents the results after applying the criteria used in the Z0011 trial. Giuliano et al. selected patients with clinical T1 or T2, N0, M0 breast cancer and one or two positive SLNs. Applying these criteria 90% (150/166) of patients would have avoided an ALND but only 7/59 (sensitivity 12%) of the patients with positive non-SLNs would have received a cALND. Apart from the prediction of positive non-SLNs, we also calculated sensitivity and specificity for the prediction of more than 3 positive lymph nodes. We regarded more than 3 positive lymph nodes as ‘bulky disease’ as it also changes stage of disease. According to the Z0011 criteria only 20% of the patients with bulky disease would have had a cALND. If we would have applied the MDA 27% cut-off, 93% of these patients would have had a cALND. In the second cohort of 114 patients we effectively used the 27% cut-off to decide on whether or not to proceed with a cALND. A total of 62/114 patients (54%) had an MDA score ≤ 27%. Applying the Giuliano criteria 90% (103/114) of the patients would avoid an ALND. In reality 68% of our patients had no cALND, clearly more then by strictly using the score. Of the 52 patients with an MDA score > 27, 16 patients (30.7%) did not get an ALND (17% of total). 3 Patients had node micro metastasis, 7 patients were more than 70 years old whereof 6 more than 75 years. 2 Patients had only a marginally elevated MDA score namely 29 and in 6 cases the reason was probably patient preference. 3 Patients (5%) with a MDA score ≤ 27% did get a cALND (2.6% of total). So in 19 patients (16% of total) the cut-off was not decisive. In 64% of the cALND no positive non-SLN’s was found. In 7 cases the cALND showed ≥ 4 positive nodes and led to an upgrading of the disease status. These 7 cases had 2 or less positive SLN’s and would not have had a cALND according to the Giuliano criteria.

**Table I:** Demographic and Clinical characteristics

Characteristics		Population 1 (n = 166)	Population 2 (n = 114)	P-value
**Age**, years				**0,002**
	mean	56,2	60,8	
Tumoursize, mm				**0,012**
	mean (min,max)	22,10 (3,80)	24,59 (5,110)	
T stage, no. (%)				**0,86**
	T1-T2	158 (95,2)	109 (95,6)	
	T3-T4	8 (4,8)	5 (4,4)	
**Grade**, no. (%)				**0,008**
	G1	15 (9,0)	22 (19,3)	
	G2	99 (59,6)	71 (62,3)	
	G3	52 (31,3)	21 (18,4)	
Estrogen Rec, no. (%)				0,1
	ER +	146 (88,0)	107 (93,9)	
	ER -	20 (12,0)	7 (6,1)	
Progesteron Rec, no. (%)				0,3
	PR +	135 (81,3)	98 (86,0)	
	Pr -	31 (18,7)	16 (14,0)	
LVI*, no. (%)				0,54
	pos	92 (55,4)	59 (51,8)	
	neg	74 (44,6)	55 (48,2)	
Total no. Pos nodes, no. (%)				N/A
	1	94 (56,6)	69 (60,5)	
	2	31 (18,7)	28 (24,6)	
	> 3	41 (24,7)	17 (14,9)	
Total no. SLNs**				**<0.0001**
	Mean	1,84	3,11	
Total no positive SLNs**				**0,039**
	Mean	1,24	1,42	
Size nodal metastasis				0,088
	Micro, no. (%)	55 (33,1)	27 (23,7)	
	Macro, no. (%)	111 (66,9)	87 (76,3)	
	Median size, mm	5,4	4,5	
Guiliano Crit., no. (%)				
	Yes	150 (90%)	103 (90%)	
	No	16 (10%)	11 (10%)	

P-value: chi-square test (categorical data) or T-test (continuous data) *LVI: lymphovascular invasion **SLNs: sentinel lymph nodes

**Table II:** Performance of the MD Anderson nomogram versus Giuliano criteria

Cut-off criterion	Number cALND saved	Any positive NSN left				>3 positive NSN left			
		Sens	Spec	PPV	NPV	Sens	Spec	PPV	NPV
MDA 27	31%	88%	41%	45%	86%	93%	36%	24%	96%
		52/59	44/107	52/115	44/51	28/30	49/136	28/115	49/51
T1-T2 / 1-2 pos SN (criteria Giuliano)	90%	12%	92%	44%	65%	20%	93%	37,5%	84%
		7/59	98/107	7/16	98/150	6/30	126/136	6/16	126/150

(cALND = completion axillary lymph node dissection, NSN = non-sentinel node, Sens = sensitivity, Spec = specificity, PPV = positive predictive value, NPV = Negative predictive value

## Discussion

The two populations, patients operated between 2002-2011 and 2012- 2015 are comparable. The significant differences in age and tumour grade 3 were unexpected and probably due to the small size of the cohorts (n1= 166, n2= 114). Significantly more SLNs were removed between 2012 and 2015 when the MDA nomogram was effectively used, compared to the first population. The number of SLNs removed affects the risk calculation by the MD Anderson nomogram. The more negative SLNs are removed, the lower the resulting calculated risk for positive non-SLNs. This knowledge probably made surgeons remove more SLNs to lower a patient’s risk for a cALND. This also explains the higher mean of the number of positive sentinels and the higher number of patients with a risk of nonSLNs below 27% in the second cohort. Several models have been developed to predict non–SLN metastases. The model we used was developed by the MD Anderson cancer centre. It is based on 7 clinicopathological variables including the maximal size of the lymph node metastasis as a continuous variable. The performance of the nomogram has been evaluated with respect to discrimination and calibration. The nomogram appeared to be reliable when applied to an internal (AUC= 0.74) and an external cohort (AUC 0.80). The average difference between predicted and calibrated probabilities was 1.4% with a maximum of 3.9% (Mittendorf et al., 2012). Zhu et al. performed a meta-analysis to evaluate 6 different nomograms. Combined data of 4 studies showed an AUC of 0.71 for the MD Anderson nomogram, suggesting a stable discriminative capability in different populations (Zhu et al., 2013). Still, the performance of a nomogram varies between populations and internal validation is needed (van den Hoven et al., 2015). A very recently published nomogram by van den Hoven et al. (2016), based on a population comparable to our Belgian population, was validated in our population. ROC analysis shows an AUC of 0.67, the 27% cut-off has a sensitivity of 74.6% and a specificity of 53%. With an AUC of 0.76 we considered the MD Anderson nomogram to be reliable in our population. The optimal cut-off of 34% has a sensitivity of 86% and a specificity of 57%. We did not use the 34% cut-off because our cohort of 166 patients is too small and results would be data driven. The discriminatory cut-off we used (27%) is based on the Z0011 trial. In the cALND group of the Z0011 trial 27.3% of the patients had additional nodal metastases. The amount of patients with residual positive nodes in their ‘SNB-alone’ group is assumed to be similar. At a median followup time of 6.3 years, the SNB-alone group and the ALND group showed no statistically significant differences in local recurrence (1.8% vs. 3.6%, P = 0.11) or regional recurrence (0.9% vs. 0.5%, p = 0.45). The same applied to the overall survival or disease-free survival. We thus regarded the 27% cutoff as a surrogate definition of a low risk population for which the results of Z0011 show that it is safe to omit a cALND. This safety also depends on whether similar adjuvant treatment is used. In the Z11 trial nearly all patients received systemic treatment and radiotherapy was given to high tangent fields in 50% of patients, covering more of the axilla than in standard radiotherapy (Reznik et al., 2005; Jagsi et al., 2014). One might question whether the 27% cut-off avoids enough cALNDs in order to have sufficient practice changing effect. In our study this approach proved to be practice changing. Although we take minimal risk to leave disease behind (2/166 patients >3 positive nodes), 30.7 % in the first cohort and 54.4 % of the patients in the second cohort did not receive a cALND. The Z0011 criteria would have had more impact, omitting 90% of the cALND. This approach clearly leaves more disease behind. Whether this difference in residual tumour leads to a significant difference in outcome remains unanswered in our study.

Long-term prospective follow-up data from breast centres omitting cALND will show whether the results of the Z0011 trial are confirmed. If confirmed a cut-off risk to perform a cALND can be abandoned at that time. In fact, one can question the role of the sentinel node procedure and indeed the SOUND trial launched in 2012 (Gentilini et al., 2012) compares SNB with no axillary surgery when preoperative axillary ultrasonography is negative. Patients with T1-T2 breast cancer receiving breast conserving surgery and adjuvant radiotherapy are eligible. Preoperative axillary ultrasound is in many centres already a standard staging examination. Trial results are awaited. The AMAROS trial (Donker et al., 2014) gave credit to another treatment option for patients with a positive sentinel. Radiotherapy was as effective as cALND while causing less lymphedema. One might select lower risk patients for omitting cALND and higher risk patients for axillary radiotherapy. Lymphedema is the most disabling side effect of axillary dissection and has an important impact on quality of life. A systematic review by Goker et al. (Goker et al., 2013) showed a 28% overall incidence of self-reported breast cancer related lymphedema. Lymphedema is decreased although not eliminated by the sentinel node procedure. (Mansel et al., 2006; Gebreurs et al., 2015) Our 27% cut-off risk of leaving metastatic nodes behind mirrors the 28% risk for lymphedema that offers a well-balanced risk-benefit based medical decision tool whether or not to proceed with a cALND. In conclusion, in our population it was possible to select patients who can be spared a cALND based on the histological criteria in the MD Anderson model with minimal risk of residual disease in the axilla. The impact of this strategy to select women for cALND according the MD Anderson nomogram with cut-off at 27% risk for positive non-SLNs on outcome is awaited. We expect a paradigm shift in the surgical management of breast cancer in the coming years. The first moves to abandoning axillary clearance have already been taken mainly driven by an immediate beneficial result on surgical morbidity.
